# Gamification as an educational tool to address antimicrobial resistance: a systematic review

**DOI:** 10.1093/jacamr/dlad130

**Published:** 2023-12-11

**Authors:** Avis A Nowbuth, Akwi W Asombang, Khuder Alaboud, Célia Souque, Butros M Dahu, Kyrtania Pather, Monica M Mwanza, Sameen Lotfi, Vikram S Parmar

**Affiliations:** Department of Neuromedicine and Movement Sciences, Norwegian University of Science and Technology (NTNU), Trondheim, Norway; Department of Research, Pan-African Organization for Health Education and Research (POHER), Manchester, MO, USA; Department of Research, Pan-African Organization for Health Education and Research (POHER), Manchester, MO, USA; Division of Gastroenterology, Massachusetts General Hospital (MGH), Harvard Medical School, Boston, MA, USA; Institute for Data Science and Informatics, University of Missouri, Columbia, MO, USA; Department of Health Management and Informatics, University of Missouri, Columbia, MO, USA; Department of Biomedical Informatics, Harvard Medical School, Boston, MA, USA; Laboratory of Systems Pharmacology, Harvard Medical School, Boston, MA 02115, USA; Institute for Data Science and Informatics, University of Missouri, Columbia, MO, USA; Department of Health Management and Informatics, University of Missouri, Columbia, MO, USA; Department of Family Medicine and Primary Care, University of the Witwatersrand, Johannesburg, South Africa; Department of Research, Pan-African Organization for Health Education and Research (POHER), Manchester, MO, USA; Department of Research, Pan-African Organization for Health Education and Research (POHER), Manchester, MO, USA; Department of Neuromedicine and Movement Sciences, Norwegian University of Science and Technology (NTNU), Trondheim, Norway

## Abstract

**Background:**

Antimicrobial resistance (AMR) poses a serious threat to global healthcare, and inadequate education has been identified as a major challenge by the WHO. The human , animal and agricultural sectors contribute to the emergence of AMR. Gamification has emerged as an innovative tool to improve knowledge and change behaviours. Our study provides an overview of the literature on existing games in prescribers’ education across the One Health sectors, with a particular focus on the impact of gamification on learning.

**Methods:**

Using the PRISMA guidelines, we searched Cochrane, PubMed, Scopus and Google Scholar for articles related to gamification for future prescribers of antimicrobials from inception until 28 March 2023. Retrieval and screening of articles was done using a structured search protocol with strict inclusion/exclusion criteria.

**Results:**

A total of 120 articles were retrieved, of which 6 articles met the inclusion criteria for final analysis. High-income countries had the most studies, with one global study incorporating low- to middle-income countries. All games were evaluated in the human sector. Board and card games, featuring scoring and point systems, were the most prevalent game types. Most games focused on improving knowledge and prescribing behaviours of medical students, with bacteria or antibiotics as the only content. All studies highlighted the significant potential of gamification in mitigating AMR, promoting antimicrobial stewardship, and improving retention of information compared with conventional lectures.

**Conclusions:**

Our review found an absence of studies in the animal and environmental sectors, disproportionately focused on medical students with questionable sample size, inadequate assessment of game content and effectiveness, and opportunities for game developers.

## Introduction

Antimicrobial resistance (AMR) is an urgent global health crisis where antimicrobials no longer effectively treat infections, leading to increased morbidity, mortality and economic burdens worldwide.^1^ In 2022, a study found that 4.95 million deaths are associated with bacterial AMR, shedding light on the burden in 2019.^2,3^ Furthermore, there are new studies that delve into the evolving concept of AMR in the One Health context, which recognizes the interdependence of human, animal and environmental health.^4^ AMR affects 12 of the 17 sustainable development goals (SDGs),^5,6^ and is substantially less published on in low- to lower-middle-income countries (LLMICs).^7,8^ The SDGs aim to anchor health in development, recognizing that good health depends on and contributes to other development goals, underpinning social justice, economic prosperity and environmental protection.^4,5^ The WHO Global Action Plan identifies lack of training and education as a core contributor to AMR, and innovative tools need to be developed to address this issue.^9,10^ Education and awareness about AMR is crucial for ensuring responsible use and preventing the development of resistance.^10^ Being aware of AMR is not enough to change prescribing behaviour; however, better knowledge on prescribing practices has the opportunity to change behaviours.^11,12^ Inappropriate practices among prescribers constitute one of the primary drivers of AMR, as seen with overprescribing, where antibiotics are prescribed for viral infections—a common practice that contributes to the development of AMR, and inappropriate prescribing such as the use of the wrong type of antibiotic or incorrect dosage, further exacerbates the issue.^13,14^ As the misuse and overuse of antimicrobial agents continue to fuel the emergence and spread of drug-resistant pathogens, addressing AMR requires innovative approaches to enhance prescribers’ practices and ensure appropriate behaviours.^15–18^ While AMR is lacking in curricula in general, efforts are being made to introduce AMR to existing curricula and develop new curricula that promote AMR awareness and education.^15,19–21^ Gamification, the use of game elements in non-game activities to increase user engagement,^22^ and serious games, games created to serve educational, training or informative objectives while maintaining engaging and immersive gameplay,^23^ have emerged as a potential educational tool to educate professionals on various health topics.^16,24^ Traditional lectures are a good source of information for many students and provide foundational knowledge,^25–27^ and gamification has been proposed as a complementary tool owing to the variety of learning styles of students.^28^ Gamification has been successfully implemented in various contexts such as the intellectual property (IP) game, IntangAbility, proving effective in teaching IP law.^29^ By integrating game elements into educational interventions, gamification has the potential to enhance learning outcomes,^30^ promote behaviour change^31^ and foster active participation^32^ in addressing AMR.^16^ However, despite the growing popularity of gamified approaches, there is a need to evaluate the effectiveness, scope and characteristics of gamification designed to assess education on AMR. Recognizing the potential of gamification in addressing AMR, this systematic review aims to examine the existing body of literature on games that assess educational interventions related to AMR. By evaluating the gamification of AMR as an educational tool, this study aims to shed light on the current state of the field and pave the way for the design and implementation of evidence-based, effective and impactful gamified tools for education for future prescribers of antimicrobials in the One Health context.

## Methods

### Search strategy

**Figure 1.**
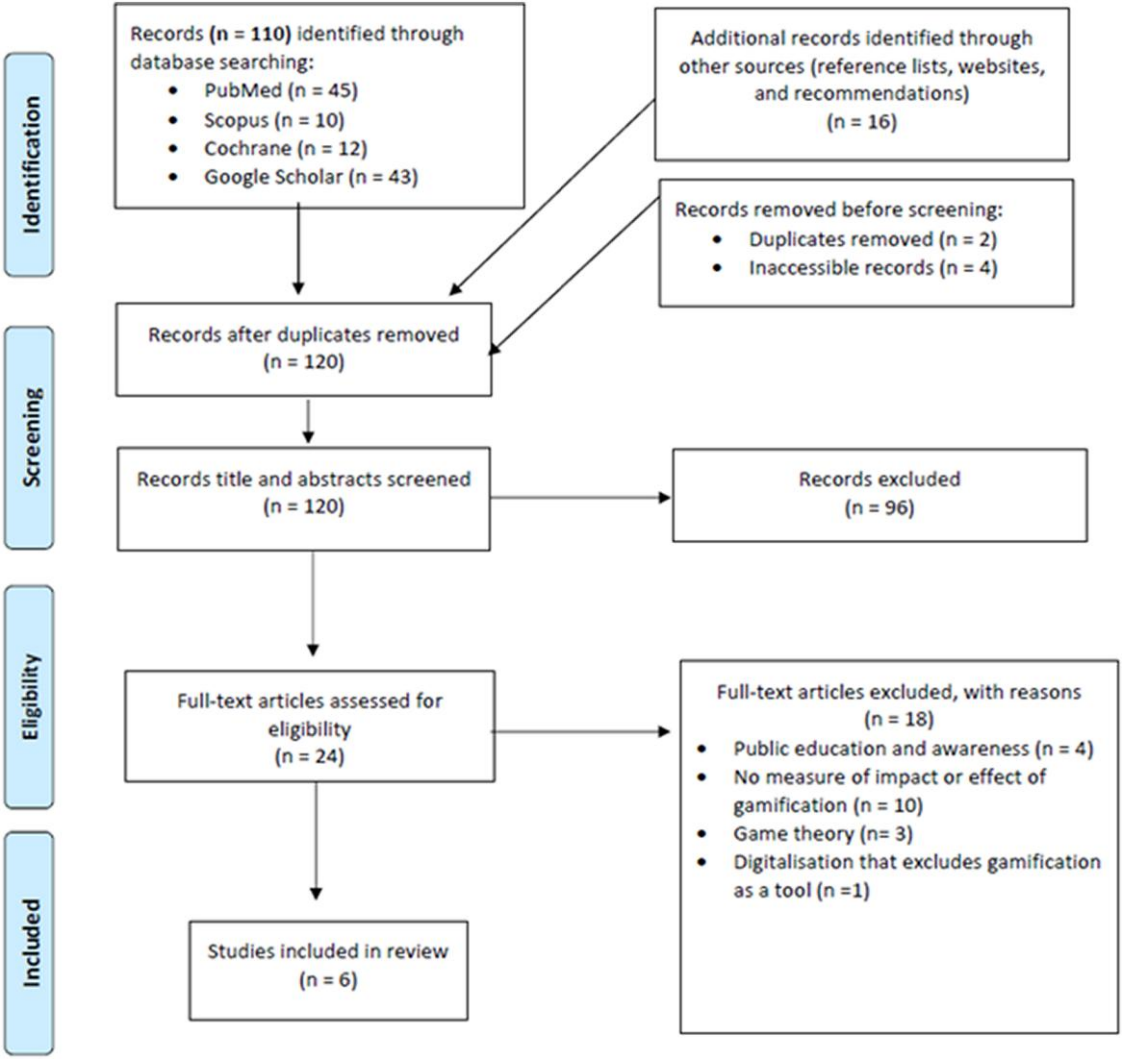
PRISMA flow chart illustrating the study selection process on gamification of AMR.

A systematic review was performed in accordance with Preferred Reporting Items for Systematic Reviews and Meta-Analysis (PRISMA) (Figure 1).^33,34^ A search strategy was developed using PubMed (Table S1, available as Supplementary data at *JAC-AMR* Online) and was fixed across all databases and grey literature. The search terms (Table 1) were used to search for literature for all three sectors such as human, animal and environment in PubMed, Scopus, Cochrane and Google Scholar. Various spellings of the search terms were considered. A total of 110 articles were identified from the four databases. An additional 10 articles were sourced from reference lists, websites and recommendations. Two articles were found to be duplicates and removed, and four articles were not accessible. No limitations on publication dates were set. Literature search began on 28 March 2023, and finished on 29 March 2023. The articles were divided, allowing for six members of the study team to independently review articles for inclusion in the analysis.

**Table 1.** Search string

Scope	String
**Gamification**	(gamif* OR games OR gameplay OR gamelike OR gamebased OR gaming OR videogam* OR edugam* OR contest)
**Education**	(medical educat* OR medical train* OR medical field training OR medical school* OR medical intern* OR medical residen* OR clinical education* OR clinical train*)
**Students**	(dental stud* OR pharmacy edu* OR nursing edu*)
	(veterin* OR animal science*)
	(agricult* OR farmers* OR environmental)
**AMR**	antimicrobial resistance OR AMR OR antimicrobial drug resist* OR drug resist* OR microbial resist* OR antibiotic resist*

### Inclusion and exclusion criteria

Full-text articles addressing the use of gamification or game elements in education for future prescribers addressing AMR were used for the review. Future health professionals were defined as students who will be responsible for prescribing antimicrobials such as medical, dental, veterinary and agricultural students. We excluded studies that (i) were targeted to the public, patients’ education or students not classified as future prescribers; (ii) did not include the effect of the intervention; (iii) only mentioned gamification but did not assess the impact; (iv) only mentioned digitalization of information; (v) mentioned game theory but did not employ game elements; and (vi) were not written in English. There were no limitations on the types of games assessed, nor timeframe for our search since game elements were used in studies before ‘gamification’ was an official term.^31^

### Study selection

A.A.N. and K.A. formatted REDCap^35,36^ and uploaded the articles used for the review. Articles were initially screened independently by seven reviewers (M.M.M., C.S., K.A., K.P., B.M.D., S.L. and A.A.N.) to determine eligibility. Each article was reviewed by at least two reviewers and conflicts were resolved by a third author. All authors then read the full text of all eligible articles to determine eligibility for inclusion. In cases of uncertainty, articles were discussed and independently screened by senior authors (V.S.P. and A.W.A.).

### Data extraction and quality assessment

The research questions were adapted to an extraction form using REDCap.^35,36^ The data extraction was independently done by M.M.M., C.S., K.A., K.P., B.D. and S.L., and verified by A.A.N. Articles that met the inclusion criteria and reported the impact of the intervention in the form of a game were included in the review. The data extracted from the articles included general information: author, year of publication, country/site, publication source and channel; geographic distribution and prevalence of articles; content of the game (AMR, antimicrobial stewardship (AMS), infectious disease (ID), clinical microbiology (CM); context of the study: human, animal, environmental or a combination of these before that was identified as a One Health paper; intervention: game format used (board game, card game, online etc.) intervention: game elements used (points, scoring, roleplay etc.); benefits and limitations of the intervention; aim of the game: knowledge, attitudes, behaviour change, entertainment etc. and key messages (Table S2a). Papers that were included for full-text review underwent a modified quality assessment. A set of closed questions were used to evaluate the relevance and quality of the article’s contents. The assessment questions were modified to fit the research questions,^32,37^ and are described in Table S2b. Scores were allocated to the included articles. QA1 scored 1 if the paper provided details about the game elements used, such as the use of points, storytelling, scoring. QA5 was subdivided considering the CORE 2022^38^ and the Journal Citation Report (JCR) 2022.

## Results

The initial search identified 110 articles [PubMed (*n* = 45), Cochrane (*n* = 12), Scopus (*n* = 10) and Google Scholar (*n* = 43)] from inception of the database to March 2023. An additional 10 articles were identified through searching reference lists, websites and recommendations. Only two duplicates were found and removed before screening. Four articles were not retrievable. A total of 120 articles were screened for eligibility based on the title and abstract contents. Overall, 96 articles were excluded due to non-relevance. Twenty-four articles were assessed for eligibility, with six articles meeting the inclusion criteria for this review. Table 2 summarizes the characteristics of the analysed articles; a full list of the included articles and breakdown is provided in Table S3.

**Table 2.** Characteristics of the articles included in this review

CHARACTERISTIC	Article # 3	Article # 56	Article # 63	Article # 88	Article # 93	Article # 98
**A**uthor	Ghelfenstein-Ferreira *et al*.	^a^Castro-Sánchez *et al*.	Davies	Valente *et al*.	Tsopra *et al*.	Ashiru-Oredope *et al*.
**Y**ear published	2021	2019	2020	2009	2020	2022
Location	Paris, France	London, UK	UK	Porto Alegre, Brazil	France	Global
Type of article	Original paper	Original paper	Short communication	Original paper		
**J**ournal	*Journal of Microbiology and Biology Education* (JMBE)	*Journal of Medical Internet research* (JMIR)	*Medical Science Educator*	*Medical Teacher*	*International Journal of Medical Informatics* (IJMI)	*MDPI Antibiotics*
One-time settings	Education evening (game night)	Workshop at a conference (International Summit on Serious Health Games)	One-off game session with additional (optional) tutorial for first-year medical students			
**G**ame(s)	Bacteria Game, KROBS and Dawaa	On call: Antibiotics	Antibiotic Top Trumps	No name	AntibioGame^®^	No name
**S**ample size	15	29	36	78	57	74
Sector of study	Human health	Human health	Human health	Human health	Human health	Human health
Game content	CM, antibiotics (20 bacteria’s traits, 20 microorganisms’ traits and means of transmission, and the usage of antibiotics)	AMS, behaviour (promoting superior antimicrobial practices while acknowledging that these practices’ ideal attributes may depend on a range of interrelated professional, clinical and organizational circumstances as well as patient expectations)	CM, antibiotics (learning about various Gram-stained microorganisms and what medications can be used to combat them)	CM, antibiotics (basic microbiology and antibiotics’ mode of action)	AMS, prescribing (public health interventions—patient interventions, antibiotic prescription and naming)	AMR, AMS, infection prevention and control, antibiotics: (1) Introduction to AMR and AMS, (2) Appropriate use of antimicrobial agents, (3) Infection prevention and control and (4) Stewardship and surveillance
**S**tudent type	Medical residents specializing in ID or CM	Consultant physicians from different clinical specialties; doctoral and postdoctoral researchers with projects focused on simulation, games, or virtual environments; AMR researchers and clinicians; experts in digital intervention implementation; games developers; behavioural researchers with interests in game-based interventions	First-year medical students	Medical and pharmacy students	Medical students having completed 2 years of medical school	Out of 74 respondents, only 7 were students
**B**enefits	Comment: significant educational contribution, favourable and meaningful positive interactions between participants. Education: increase in scores during the evening regarding questions addressed by the games.	Comment: fosters excellent antimicrobial behaviours—multidisciplinary approach and includes management of patient expectations.	Education: little difference before and after playing the game. Themes were fun, content of the cards were helpful, and pictures were nice, a fun way to apply knowledge, a good revision aid.	Education: significant increase in the number of right answers, decrease in number of unknown answers. Comment: interesting, with clear design and improved knowledge about the subject, important way of enhancing learning, literature appropriate. Valuable for improvement of intellectual skills.	Education: attractive, fun, and appropriate for learning about antibiotics. A good revision aid.	Comment: the game was entertaining and enriching, interesting and straightforward. Incorporates the One Health approach partially, and both HICs and LMICs.
**L**imitations	Bacteria Game—independent gameplay or under supervision of CM; KROBS—independent gameplay or supervision of CM; Dawaa—needs supervision of ID specialist. Low number of participants (15) does not conclude that the use of games does not have an impact on learning in general. One social event does not mean that the increase in scores is attributable to the game. Study pool are students who already have knowledge in ID and CM.	Does not aim to teach about specific antibiotics, appropriateness, or effectiveness in the treatment of an infection	Education: fun but not useful to learn, too fast paced, wasn’t paying attention to card details, rather just the stats of the card. Suggestion that playing with cards at intervals over weeks could result in better retention of information.	Education: does not provide acquisition of practical/manual skills. Independent game, no need for supervision.	Education: there is a limited bank of questions (10 cases only), the topic did not extend to other specialities, topics related to AMR such as mechanisms of bacterial resistance were excluded, medical context was only for GP office and excluded the management opportunities for things like ICU.	Comment: the demonstration was rushed, therefore unable to get a real feel of the game, it was played for a very short time. Cross-talking among participants made it difficult for others to respond and the facilitators talked too much.
**T**ype of game	Board game, 2 card games	Mobile case-based game	Card game	Board game	Online game (case-based game)—serious game—interactive	Online board game
**G**ame elements	Point scoring	Story roleplay	Scoring	Leaderboard	Challenge, mascot (Antibioman), avatar with badges, hints, feedback, rewards, points, level and progress bars, leaderboard, immersion in the real world	Points
**A**im of the game	Entertainment, improve knowledge, address prescribing practices	Entertainment, address prescribing practices, change behaviour	Improve knowledge, change behaviour	Improve knowledge	Address prescribing practices, change behaviour	Improve knowledge change behaviour
**D**escription of the intervention	The board game and 2 card games were played at a game night for residents.	The game resembles clinical practice: there are virtual patients that present with a condition, and the student must use and employ diagnostic skills and optimal behaviours that one should be familiar with based on the established antibiotic guidelines in the country (UK).	The game resembles an existing game called Top Trumps; it has been tailored to information related to antibiotics. The cards had details about commonly used antibiotics with scores against the microorganisms, toxicity and administration routes. It also had an additional fun/useful fact.	The game is an illustrated board game. Players must move their token across the board game, they pass over squares in which there are antibiotic name, and ‘pick up a card’ squares. The cards detail a bacterium and entails information on whether it is susceptible/resistant or intermediate resistant to the antibiotic card. In addition, there are also cards where there are textbook questions. The player who picks up the card has to answer the questions.	This game is a case-based game for teaching students about antibiotics in primary care. The player plays the role of doctor and solves the case using clinical reasoning.	The game is an online board game similar to the ‘snakes and ladders’ concept.
**G**ameplay evaluation	Played for 30 min	2 hour workshop. On call: Antibiotics developed in 2015, with 4000 downloads. Not an evaluation of the game *per se*; but rather a workshop setting to debate the limitations and gaps.	Played for 10 min; pre-test and post-test with additional optional lecture.	Between 40 and 90 min; pre-test and post-test with additional lecture given a week prior to the intervention.	Each session began with a presentation of the game. Three clinical cases were played by the students before an evaluation and comparison of the scores took place.	The online game was played by over 100 students in 23 different countries on two occasions (August 2021 and November 2021) and was played using Zoom. The game lasted 45 min. Only 74 participants completed the feedback form upon completion of the intervention, of which, only 7 participants who responded were students.

^a^Castro-Sánchez *et al*.^40^ has been highlighted here as a study in which the intervention was evaluated, with limited evaluation of the game itself.

### Study characteristics

A total of six articles evaluated games addressing AMR in the human healthcare sector. There were five original papers, and one short communication paper.

The articles were distributed throughout various journals and publication channels, of different ranking. Articles were published in *Journal of Microbiology and Biology Education* (JMBE),^39^  *Journal of Medical Internet Research* (JMIR),^40^  *Medical Science Educator*,^17^  *Medical Teacher*,^41^  *International Journal of Medical Informatics* (IJMI)^42^ and *MDPI Antibiotics*.^43^ No articles were published as conference abstracts or symposia. The ranking of the publication sources was considered to investigate the reach of the articles. The majority of the articles were ranked as Q2, Q3 and Q4.

**Figure 2.**
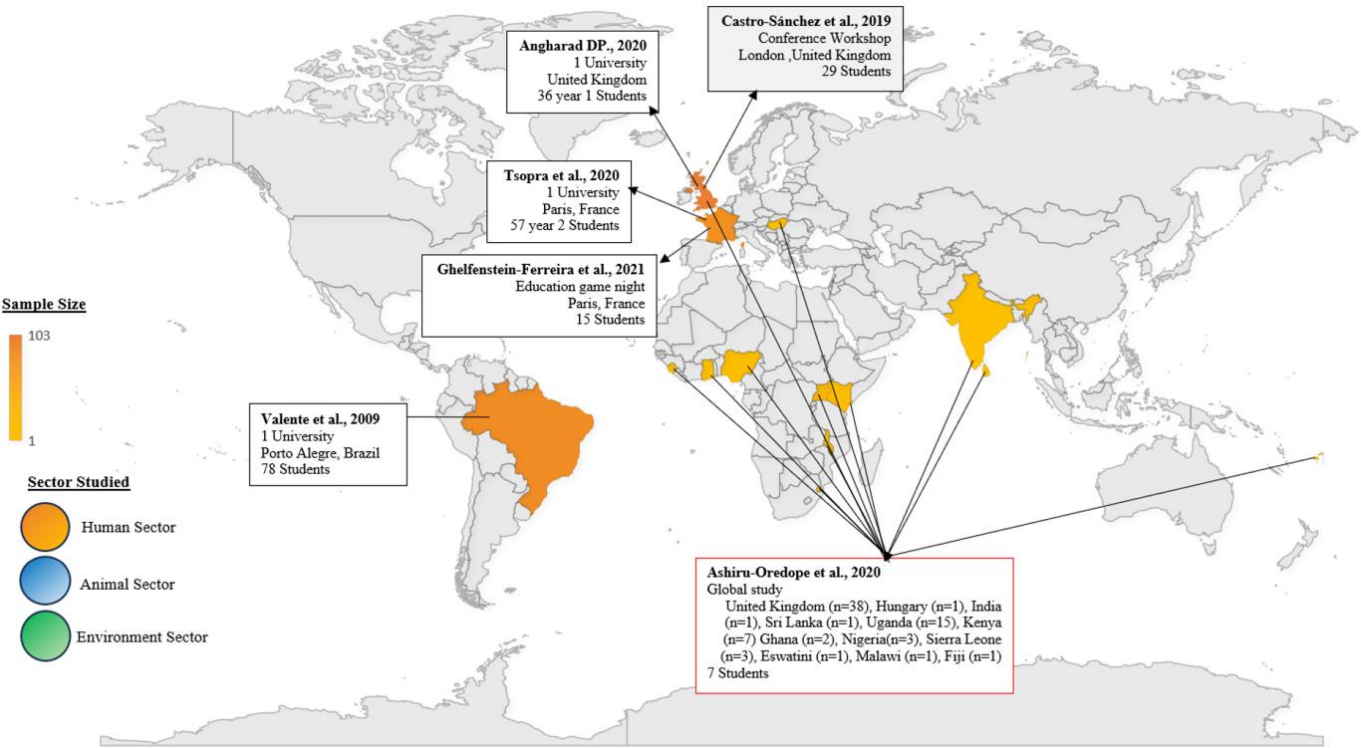
Geographical distribution of participants and sector studied evaluating gamification studies on AMR included in this systematic review.

The countries that reported numerical and statistically relevant results are shown in Figure 2. The publication trend highlights the novelty of the topic of gamification, as the majority of the studies were between 2019 and 2022, with one game piloted in 2009. Four of the six studies (67%) occurred in high-income countries (HICs) (France and the UK).^34^ Ghelfenstein-Ferreira *et al*.^39^ and Tsopra *et al*.^42^ conducted their studies in France, with a sample size of 15 participants, and 57 participants, respectively. Davies^44^ and Castro-Sánchez *et al*.^40^ conducted their studies in the UK, with a sample size of 36 and 29 participants, respectively, partaking in the intervention. Valente *et al*.^41^ surveyed one university in Brazil amongst 78 participants. Ashiru-Oredope *et al*.^43^ conducted an online study from the UK, which included a global representation of 13 countries with 74 responses [UK (*n* = 38), Hungary (*n* = 1), India (*n* = 1), Sri Lanka (*n* = 1), Uganda (*n* = 15), Kenya (*n* = 1), Ghana (*n* = 1), Nigeria (*n* = 1), Sierra Leone (*n* = 1), Eswatini (*n* = 1), Malawi (*n* = 1) and Fiji (*n* = 1)].

Each study had assessed at least one game, with multiple topics being highlighted. Among the studies assessed, three out six studies (50%) highlighted games that aimed to educate players about bacteria and antibiotics, including medication names and modes of action against bacteria.^29,31,35^ However, there was relatively less emphasis on AMS, with only two games (33%) addressing this crucial aspect.^30,33^ One study, by Castro-Sanchez *et al*.,^40^ explored patient expectations, the role of behaviours in stewardship, and the multidisciplinary roles of professionals to mitigate AMR by evaluating the setting in which games are deployed. Another study, by Tsopra *et al*.,^42^ focused on the role of prescribing practices of healthcare professionals in reducing AMR. Only one game^43^ introduced the concept of AMR, encompassing aspects such as AMR and stewardship introduction, proper application of antibiotics, prevention and control of infections, as well as stewardship and surveillance. No reviewed games included other microorganisms or addressed the interlinkage between human, animal and environmental sectors in the context of AMR.

Most of the studies in the healthcare setting were conducted on medical students. Each of these studies employed varied definitions and incorporated ‘students’ within this framework, encompassing those specializing in infectious diseases, clinical microbiology, pharmacy and medical studies. Only three studies focused on medical students specifically, while others included them alongside specialists and physicians. No studies were found for veterinary or agricultural students. Additionally, there are no games that addressed AMR across all sectors.

Ghelfenstein-Ferreira *et al*.^39^ evaluated two existing card games and a board game, while other studies evaluated their own developed game: one mobile case-based game,^40^ one card game,^44^ one board game,^41^ one online case-based interactive game^42^ and one online board game.^43^ Physical board games and card games (5/8; 62.5%) were the most popular type of game to be employed as information tools for students. Ashiru-Oredope *et al*.^43^ evaluated an online board game. Two games were based on case-based clinical scenarios on an online platform.^40,42^ The games were evaluated in isolated, one-time settings such as at a planned educational evening (game night) for residents,^39^ at workshops at conferences^40^ and university settings.^41^

The game elements employed in the reviewed AMR games exhibited variation across different games. However, a notable trend emerged in that most of these games primarily utilized a common concept of points and scoring as the main incentive to encourage user engagement and progression within the game. This observation highlights that, while gamification as a concept encompasses a wide range of elements, such as leaderboards, challenges and feedback mechanisms, these specific approaches were less frequently employed in the context of AMR games, as indicated in Table 2. One game^42^ was an exception as it incorporated more than two game elements and mechanics. This game employed points, rewards, storylines, roleplay, progress bars and leaderboards through an online platform, creating a multifaceted gaming experience. It is also noteworthy that card and board games primarily made use of scores and points as their primary gaming elements, while online games had the flexibility to incorporate a variety of game mechanics. For instance, the online games, even in the context of clinical case scenarios, included animated characters to represent the users, enhancing the user experience.

The benefits and limitations of each game are summarized in Table S3. Most studies evaluated the students’ knowledge prior to the intervention and post intervention.^41,42,44^ It is important to note that the ‘post-tests’ all occurred immediately after the game, and not after a longer period. The post-tests showed an increase in correct answers and increase in scores after the intervention (game),^41,42,44^ and some studies included a qualitative component evaluating the game entertainment, usefulness and thoughts if included in the curricula. Most studies showed that the games were considered ‘fun and enjoyable’, the ‘pictures were nice’, and that it could be a ‘valuable intervention for improvement of intellectual skills, improve knowledge and enhance leanings’. Davies’s findings^44^ showed that while students enjoyed the game, it would be better suited as a revision aid compared with a standalone lecture. Some of the limitations across all games were that there was not a large enough sample size to correlate the retention of memory with the game, and a suggestion was to introduce gameplay across several weeks and assess the effects of the game over a period. Some of the games had to be played under supervision of an ID specialist or clinical microbiologists^39^ to get a better understanding of the game. Davies’s feedback^44^ included that the game was fun but not useful to learn, it was too fast paced, and the details of the microorganism or antibiotics were not the main point of attraction, rather the focus was on how ‘good’ the statistics on the card were. Other challenges that the studies highlighted include the need for tutorials for students in early years of medical school, poor sample size to make claims of whether the intervention was considered successful in retaining knowledge, games as a potential distraction from learning, and lack of detailed accounts of replicable implementation, adoption or evaluation.

The aim of all the games, across all six studies, had very similar overall objectives, namely to (i) improve knowledge, (ii) provide entertainment, (ii) address prescribing practices and (iv) change behaviours. Ghelfenstein-Ferreira *et al*. ^39^ reported positive outcomes, with participants demonstrating improvements in their knowledge of ID and CM as well as progress in their professional training. Students responded positively to the study, considering the information obtained from the game to be valuable. In contrast, Davies’s study^44^ found that the students’ scores remained unchanged before and after the intervention, and although some students found the game enjoyable, they did not perceive it as a useful tool for learning. This perception was primarily influenced by the game’s fast-paced nature and the requirement of prior knowledge. Valente *et al*.^41^ observed significant improvements in knowledge, as evidenced by a higher number of correct answers and a decrease in unknown answers after the intervention. Participants praised the intervention for its clear design and its effectiveness in enhancing learning. However, Valente *et al*.^41^ also noted that the games did not provide opportunities for the acquisition of practical or manual skills. Castro-Sánchez *et al*.^40^ raised concerns about potential challenges that may arise when implementing gamified approaches. Although they recognized the potential of gamification in enhancing our understanding of AMR, they emphasized the requirement for additional research to validate its legitimacy and effectiveness when contrasted with conventional learning approaches. Tsopra *et al*.’s evaluation^42^ primarily focused on players’ reactions to the game rather than their learning abilities. The game was primarily seen as a revision aid for microbiology. Ashiru-Oredope *et al*.^43^ found that participants exhibited a positive experience and improved knowledge retention regarding AMR after playing the game. However, they noted that the game demonstration was rushed, making it difficult to assess its true potential. Additionally, participants mentioned challenges related to cross-talking among players and excessive facilitator involvement. The comments and aims of the game can be found in Table S1.

## Discussion

This systematic review is the first to examine the current evidence of existing games in the education and training of future prescribers in the healthcare setting (medical students), animal sector (veterinarians) and the environmental sector (agriculture science). The majority of the studies included in this review were both qualitative and mixed-methods studies located in HICs. All the studies reported an increase in knowledge scores upon evaluation of their interventions, except for one study where the scores remained unchanged.

Our review highlights a small number of articles that evaluate gamification of AMR, with a predominance in HICs. Furthermore, none of the games addressed the interlinkage between the three sectors: human, animal and environmental, which are all relevant in the context of AMR.

The six articles analysed were published in a variety of journals that focused on gamification, medical education and AMR. Gamification is a multidisciplinary innovative intervention that can be implemented to enhance education.^21^ The WHO highlights the urgency for investment in infrastructure and resources that provide capacity building, and specifically innovative interventions for control of AMR.^10^ Despite these three subdisciplines (human healthcare, animal healthcare and environmental sectors) being distinct fields, there are several reasons why a combination and adoption as an interdisciplinary approach is vital to mitigate the spread of AMR. Approaching AMR, gamification and education of students as a merged field provides a more comprehensive understanding of complex issues. Gamification as an educational tool for AMR can enhance knowledge and engagement among students that will better antimicrobial stewardship and prevent overuse and misuse of antimicrobials in the long term.^11,20^ Furthermore, interdisciplinary collaborations can lead to innovative solutions that may not have been possible within a single field. By merging gamification, AMR and education, research findings can be translated into educational programs and games to promote awareness, change behaviours and establish better practices.^21,22^

The majority of published papers predominantly focus on studies and interventions conducted in HICs. This observation aligns with historical patterns of LLMICs tending to receive limited attention during the implementation of new interventions.^45,46^ It is noteworthy that a single study adopts a global approach; nevertheless, due to the low participant representation, with an average of one participant per country, caution must be exercised in drawing conclusions regarding the effectiveness of the game. Since AMR is a global issue, interventions from the Global North may not be applicable or implementable for the Global South, hence it is important to create an environment for students in the Global South.^6,38,39^ Similarly, interventions should be created and tailored for students in the Global South. Low- to middle-income countries (LMICs) have implemented game-based interventions, such as a web-based trivia game designed for emergency medical technicians,^47^ and a digital game-based intervention to improve adolescent mental health in schools in India.^48^

Antibiotics and bacteria play a central role in the development and spread of AMR;^40^ however, studying bacteria addresses only one-quarter of the proposed problem of AMR, as microorganisms that contribute to the spread of AMR also include parasites, viruses and fungi.^36^ While games primarily focusing on antibiotics and bacteria address crucial aspects of AMR, it is important to acknowledge that AMR is a multidimensional issue that involves various factors beyond antibiotics and bacteria. Future game developers and researchers may consider expanding the scope of games addressing AMR to include additional aspects, such as stewardship practices, policy implications, One Health perspectives, societal behaviours, and the broader context of healthcare systems. By incorporating these additional dimensions, games can provide a more comprehensive understanding of AMR and promote a holistic approach to tackling the challenge.

Assessing the impact of games on medical students is of utmost importance, as they play a vital role as primary caregivers and are often the first point of contact for the public when it comes to prescribing antibiotics.^32^ However, it is equally important to extend the scope of assessment to include other healthcare professions, such as dentists, who also contribute significantly to the responsible use of antibiotics. One study, excluded from this review due to the absence of dentist participation, underscores the significance of antibiotics in oral healthcare.^20^ Even though medical students were the main audience, there is still a limited number of studies that evaluated the gameplay and suggests a potential gap in understanding the effectiveness of gamification amongst students.

Overall, the poor sample size and focus on the human healthcare sector is a fragmented approach highlighting the need for efforts in addressing AMR using an interdisciplinary approach to address the interconnectedness of AMR. To prevent AMR from worsening, this issue requires collaboration among medical, veterinary and agricultural sciences to promote a holistic understanding and implement effective strategies.

Physical games like board and card games are considered the most popular games. Board and card games are familiar with a greater audience, decreasing the barriers to adoption and increases the likelihood of engagement.^41,42^ However, while physical games have their advantages, they also have certain limitations: limited scalability and customization; need for physical components and repetitiveness.^41,42^ Digital games and online platforms offer unique benefits, such as scalability, customization, multimedia integration and real-time feedback/assessment.^42,43^ One of the main limitations of online and digital games is the reliance on internet access and suitable devices, and the need to maintain devices with updated versions, which may not be supported; these factors may pose limitations in areas with limited connectivity or inadequate technology resources.^44^

While there are multiple gamification opportunities available to researchers and developers, the most effective tool for information retention and change of behaviours has not yet been established. Furthermore, a notable disparity exists between student preferences for popular games and the feasibility of creating and implementing games from a research perspective.^49,50^ This discrepancy presents an opportunity for researchers to develop and introduce innovative game-based solutions in educational settings.

Landers introduced a gamified learning theory that is founded by two frameworks: (i) a framework that describes game elements that have the potential to improve learning; and (ii) a theoretical model that links learning with gamification efforts.^45,46^ This model can be used to support the link between elements and learning opportunities using attitudes and change of behaviours.^46^ The time spent on the game can be directly linked to the increase in the performance on the subject matter.

Some popular game mechanics and elements have been used in the development and feedback of the games in our review. Feedback and progression bars are a good tool to highlight areas needed for improvement by the student. When students receive feedback on their goal, they have the possibility to reinforce and refocus their learning efforts.^46^ One aspect that can help goal-directed behaviours and better participation is setting clear rules and goals, such as needing to complete 90% of the game or attendance. This game element was not used exclusively in the reviewed games; however, it has been suggested to influence learning.^46^ Assessment game elements include points, scores, badges and leaderboards. While this is a good incentive for learning, not all participants react in the same way to these elements.^47^ Further research is required to gain a comprehensive understanding of how gamification elements precisely stimulate motivation, as varying perspectives on their effectiveness persist.^51,52^ This understanding is crucial for the appropriate implementation of these game mechanics, emphasizing the need for continued investigation.

Articles addressing gamification as a tool to mitigate AMR are lacking. Of the articles reviewed, there are some concerns about the quality of the evidence presented. Few papers provide detailed descriptions of the implementation and evaluation of the games.^30^ Information is fresh in the mind right after the lecture, and one may have a better ability to recall details and concepts,^48^ therefore suggesting knowledge improvement is attributable to the game can be argued.

It would be beneficial to assess how games were implemented, tested and reported in more than one setting, and with an additional comparator,^49^ to fully assess the effect of the gamified intervention. One aspect of gamification that has not been fully explored is games as a potential distraction instead of learning.^14^ While Tsopra *et al*.^42^ had a good approach for evaluating the game, notably only the ‘reaction’ level was assessed, therefore there is a further need to evaluate the learning, behaviour and results aspects of the game, as suggested in the evaluation of training, to properly assess the effect of the game as a tool for education.^50^ Traditional lectures provide positive impact on students, promoting deontological education, and facilitating the formation of professional self-awareness and reflection;^53^ however, they also have drawbacks, namely the lack of engagement,^54^ passive learning,^55^ stagnation^55^ and inactiveness of students.^56^ With the increase in digital literacy, we need to provide innovative (gamified) ways of learning.

Different social settings can have a significant impact on game experience, memory retention and understanding of a subject. While competition can be exciting and motivating, it may also lead to increased stress and pressure.^51^ In such settings, memory retention and understanding of the subject can be influenced by the desire to outperform others, leading to enhanced focus and engagement, but also potentially impairing learning if the emphasis is solely on winning.^51,52^ Specific social settings can influence game experience and memory retention by affecting collaboration, competition, social presence, peer influence, group dynamics and cultural/contextual factors. Without understanding these influences, there is a need to consider how to create a game with more effective learning environments and how researchers and game developers can enhance the overall educational value of games.

Each of the articles focused on developing an intervention specifically designed for students with limited understanding of AMR. The findings of our review revealed that the implementation of gamification had a significant impact on the participants. At first, the positive response towards gamification can be misleading as there is a scarcity of articles and games related to AMR, and caution must be drawn when making strong conclusions justifying the use of games in education.^21^ Most of the studies used descriptive analysis, with no control groups; the effectiveness of games in curtailing AMR remains uncertain. The review also points out some challenges and limitations associated with gamification in AMR education. The use of gamification in addressing AMR, improving knowledge and changing behaviour is promising; however, these observations highlight the importance of carefully designing and implementing gamified interventions to maximize their educational impact. There is a lack of a theoretical model, and we should consider a pedagogical approach in making games more effective in delivering educational information.^53^

### Strengths and limitations

This review was conducted using a formulated protocol and used the PRISMA guidelines for conducting and reporting systematic reviews.^23,24^ All co-authors were involved in all stages of the review and each author independently performed stages from title/abstract screening to data extraction. All articles were double-checked by the corresponding author and senior author. The research team met at the beginning and end of each stage to voice concerns, ideas and discrepancies for conflicting articles. The senior authors, along with the first author, made final decisions based on discussion and agreements. All authors had full access to all the data in the study. All authors read and approved the submitted version.

As a result of the number of studies included in this systematic review, it was not possible to do a meta-analysis of the results of the available studies. The variety of interventions and variety of reporting styles limited clear categorizations of these interventions. However, this was mitigated by using gamification elements. Additionally, since many interventions reported improvements in knowledge and skills, there is a potential for publication bias.^54^ Publication bias is possible as studies with negative outcomes are less likely to be reported or accepted for publication.^54^ It is possible that more unpublished evidence may exist. Furthermore, while there are online games available, the authors could not find any articles linking to the effectiveness of the game (e.g. Pharmageddon: Bugs versus Drugs).

### Conclusions

Our review found that there is a lack of studies in the animal and environmental sectors, a disproportionate attention on medical students, a lack of interdisciplinary approach, inadequate assessment of game content and effectiveness, and potential opportunities for future game development. Of the games evaluated, no game addressed the concerns around the impact of AMR on animal health, agricultural practices and spread of AMR. Given that antimicrobials are used in animals and in agricultural practices, there is a need to explore educational interventions in these sectors as well.

Our study reveals opportunities for future research and game development that can bridge the gap between opportunities for AMR education and effective gamification interventions. Developing educational interventions that encompass human healthcare, animal and environmental sectors, and incorporating gamification and interactivity elements could enhance knowledge transfer, collaboration and the overall understanding of AMR among healthcare professionals, students and researchers. We suggest the implementation of a universal module that may be applied to tackle AMR across all domains. By incorporating gamification within the framework of digital literacy, the educational sector can benefit significantly. This approach can foster student engagement and motivation, and encourage favourable behavioural changes among students.

## Supplementary Material

dlad130_Supplementary_Data

## Supplementary data

Tables S1 to S3 are available as Supplementary data at *JAC-AMR* Online.
