# Supplementary material for: Gamification as an educational tool to address antimicrobial resistance: a systematic review
Source: JAC Antimicrob Resist. 2023 Dec 11;5(6):dlad130. doi: 10.1093/jacamr/dlad130 (PMC10712719; doi:10.1093/jacamr/dlad130)
Supplement: dlad130_Supplementary_Data [file dlad130_supplementary_data.docx]

Table S1. Search Strategy Development Gamification AMR

| **Search** | **Query** | **Results** | **Time** |
| --- | --- | --- | --- |
| #4 | Search: **(((medical educat* OR medical train* OR medical field training OR medical school* OR medical Intern* OR medical residen* OR medical student* OR dental student* OR nursing student* OR pharmacy student* OR veterinary student* OR clinical education* OR clinical train* OR clinical Intern* OR clinical residen* OR clinical clerk* OR teaching round* OR dental education* OR pharmacy education* OR pharmacy residen* OR nursing education* OR paramedics education* OR paramedic education* OR paramedical education* OR physiotherapy education* OR physiotherapist education* OR emergency medical services educat* OR curricul* OR veterinary education OR allied health personnel)) AND ((Antimicrobial Drug Resistance* OR AMR OR Antimicrobial Resistance OR antimicrobial Drug Resistances, Microbial OR microbial Resistance OR antibiotic Resistance))) AND ((((((((((gamif*) OR (gameplay)) OR (game)) OR (games)) OR (gamelike)) OR (gamebased)) OR (gaming)) OR (videogam*)) OR (edugam*)) OR (contest))** | [45](https://pubmed.ncbi.nlm.nih.gov/?term=%28%28%28medical+educat%2A+OR+medical+train%2A+OR+medical+field+training+OR+medical+school%2A+OR+medical+Intern%2A+OR+medical+residen%2A+OR+medical+student%2A+OR+dental+student%2A+OR+nursing+student%2A+OR+pharmacy+student%2A+OR+veterinary+student%2A+OR+clinical+education%2A+OR+clinical+train%2A+OR+clinical+Intern%2A+OR+clinical+residen%2A+OR+clinical+clerk%2A+OR+teaching+round%2A+OR+dental+education%2A+OR+pharmacy+education%2A+OR+pharmacy+residen%2A+OR+nursing+education%2A+OR+paramedics+education%2A+OR+paramedic+education%2A+OR+paramedical+education%2A+OR+physiotherapy+education%2A+OR+physiotherapist+education%2A+OR+emergency+medical+services+educat%2A+OR+curricul%2A+OR+veterinary+education+OR+allied+health+personnel%29%29+AND+%28%28Antimicrobial+Drug+Resistance%2A+OR+AMR+OR+Antimicrobial+Resistance+OR+antimicrobial+Drug+Resistances%2C+Microbial+OR+microbial+Resistance+OR+antibiotic+Resistance%29%29%29+AND+%28%28%28%28%28%28%28%28%28%28gamif%2A%29+OR+%28gameplay%29%29+OR+%28game%29%29+OR+%28games%29%29+OR+%28gamelike%29%29+OR+%28gamebased%29%29+OR+%28gaming%29%29+OR+%28videogam%2A%29%29+OR+%28edugam%2A%29%29+OR+%28contest%29%29&sort=) | 15:52:37 |
| #3 | Search: **(((((((((gamif*) OR (gameplay)) OR (game)) OR (games)) OR (gamelike)) OR (gamebased)) OR (gaming)) OR (videogam*)) OR (edugam*)) OR (contest)**  "gamif*"[All Fields] OR "gameplay"[All Fields] OR "game"[All Fields] OR "game s"[All Fields] OR "games"[All Fields] OR "gaming"[All Fields] OR "gamelike"[All Fields] OR "gamebased"[All Fields] OR "game s"[All Fields] OR "games"[All Fields] OR "gaming"[All Fields] OR "videogam*"[All Fields] OR "edugam*"[All Fields] OR "contest"[All Fields] OR "contestability"[All Fields] OR "contestable"[All Fields] OR "contestant"[All Fields] OR "contestants"[All Fields] OR "contestation"[All Fields] OR "contestations"[All Fields] OR "contested"[All Fields] OR "contesting"[All Fields] OR "contests"[All Fields]  **Translations**  **games:** "game's"[All Fields] OR "games"[All Fields] OR "gaming"[All Fields]  **gaming:** "game's"[All Fields] OR "games"[All Fields] OR "gaming"[All Fields]  **contest:** "contest"[All Fields] OR "contestability"[All Fields] OR "contestable"[All Fields] OR "contestant"[All Fields] OR "contestants"[All Fields] OR "contestation"[All Fields] OR "contestations"[All Fields] OR "contested"[All Fields] OR "contesting"[All Fields] OR "contests"[All Fields] | [65,555](https://pubmed.ncbi.nlm.nih.gov/?term=%28%28%28%28%28%28%28%28%28gamif%2A%29+OR+%28gameplay%29%29+OR+%28game%29%29+OR+%28games%29%29+OR+%28gamelike%29%29+OR+%28gamebased%29%29+OR+%28gaming%29%29+OR+%28videogam%2A%29%29+OR+%28edugam%2A%29%29+OR+%28contest%29&sort=) | 15:52:21 |
| #2 | Search: **(Antimicrobial Drug Resistance* OR AMR OR Antimicrobial Resistance OR antimicrobial Drug Resistances, Microbial OR microbial Resistance OR antibiotic Resistance)**  (("anti infective agents"[Pharmacological Action] OR "anti infective agents"[MeSH Terms] OR ("anti infective"[All Fields] AND "agents"[All Fields]) OR "anti infective agents"[All Fields] OR ("antimicrobial"[All Fields] AND "drug"[All Fields]) OR "antimicrobial drug"[All Fields]) AND "resistance*"[All Fields]) OR ("appl magn reson"[Journal] OR "altern med rev"[Journal] OR "amr"[All Fields]) OR ("drug resistance, microbial"[MeSH Terms] OR ("drug"[All Fields] AND "resistance"[All Fields] AND "microbial"[All Fields]) OR "microbial drug resistance"[All Fields] OR ("antimicrobial"[All Fields] AND "resistance"[All Fields]) OR "antimicrobial resistance"[All Fields]) OR (("drug resistance, microbial"[MeSH Terms] OR ("drug"[All Fields] AND "resistance"[All Fields] AND "microbial"[All Fields]) OR "microbial drug resistance"[All Fields] OR ("antimicrobial"[All Fields] AND "drug"[All Fields] AND "resistances"[All Fields]) OR "antimicrobial drug resistances"[All Fields]) AND ("microbial"[All Fields] OR "microbially"[All Fields] OR "microbials"[All Fields])) OR (("microbial"[All Fields] OR "microbially"[All Fields] OR "microbials"[All Fields]) AND ("resist"[All Fields] OR "resistance"[All Fields] OR "resistances"[All Fields] OR "resistant"[All Fields] OR "resistants"[All Fields] OR "resisted"[All Fields] OR "resistence"[All Fields] OR "resistences"[All Fields] OR "resistent"[All Fields] OR "resistibility"[All Fields] OR "resisting"[All Fields] OR "resistive"[All Fields] OR "resistively"[All Fields] OR "resistivities"[All Fields] OR "resistivity"[All Fields] OR "resists"[All Fields])) OR ("drug resistance, microbial"[MeSH Terms] OR ("drug"[All Fields] AND "resistance"[All Fields] AND "microbial"[All Fields]) OR "microbial drug resistance"[All Fields] OR ("antibiotic"[All Fields] AND "resistance"[All Fields]) OR "antibiotic resistance"[All Fields])  **Translations**  **Antimicrobial Drug:** "anti-infective agents"[Pharmacological Action] OR "anti-infective agents"[MeSH Terms] OR ("anti-infective"[All Fields] AND "agents"[All Fields]) OR "anti-infective agents"[All Fields] OR ("antimicrobial"[All Fields] AND "drug"[All Fields]) OR "antimicrobial drug"[All Fields]  **AMR:** "Appl Magn Reson"[Journal:__jid9204100] OR "Altern Med Rev"[Journal:__jid9705340] OR "amr"[All Fields]  **Antimicrobial Resistance:** "drug resistance, microbial"[MeSH Terms] OR ("drug"[All Fields] AND "resistance"[All Fields] AND "microbial"[All Fields]) OR "microbial drug resistance"[All Fields] OR ("antimicrobial"[All Fields] AND "resistance"[All Fields]) OR "antimicrobial resistance"[All Fields]  **antimicrobial Drug Resistances,:** "drug resistance, microbial"[MeSH Terms] OR ("drug"[All Fields] AND "resistance"[All Fields] AND "microbial"[All Fields]) OR "microbial drug resistance"[All Fields] OR ("antimicrobial"[All Fields] AND "drug"[All Fields] AND "resistances"[All Fields]) OR "antimicrobial drug resistances"[All Fields]  **Microbial:** "microbial"[All Fields] OR "microbially"[All Fields] OR "microbials"[All Fields]  **microbial:** "microbial"[All Fields] OR "microbially"[All Fields] OR "microbials"[All Fields]  **Resistance:** "resist"[All Fields] OR "resistance"[All Fields] OR "resistances"[All Fields] OR "resistant"[All Fields] OR "resistants"[All Fields] OR "resisted"[All Fields] OR "resistence"[All Fields] OR "resistences"[All Fields] OR "resistent"[All Fields] OR "resistibility"[All Fields] OR "resisting"[All Fields] OR "resistive"[All Fields] OR "resistively"[All Fields] OR "resistivities"[All Fields] OR "resistivity"[All Fields] OR "resists"[All Fields]  **antibiotic Resistance:** "drug resistance, microbial"[MeSH Terms] OR ("drug"[All Fields] AND "resistance"[All Fields] AND "microbial"[All Fields]) OR "microbial drug resistance"[All Fields] OR ("antibiotic"[All Fields] AND "resistance"[All Fields]) OR "antibiotic resistance"[All Fields] | [375,583](https://pubmed.ncbi.nlm.nih.gov/?term=%28Antimicrobial+Drug+Resistance%2A+OR+AMR+OR+Antimicrobial+Resistance+OR++antimicrobial+Drug+Resistances%2C+Microbial+OR+microbial+Resistance+OR+antibiotic+Resistance%29&sort=) | 15:51:35 |
| #1 | Search: **(medical educat* OR medical train* OR medical field training OR medical school* OR medical Intern* OR medical residen* OR medical student* OR dental student* OR nursing student* OR pharmacy student* OR veterinary student* OR clinical education* OR clinical train* OR clinical Intern* OR clinical residen* OR clinical clerk* OR teaching round* OR dental education* OR pharmacy education* OR pharmacy residen* OR nursing education* OR paramedics education* OR paramedic education* OR paramedical education* OR physiotherapy education* OR physiotherapist education* OR emergency medical services educat* OR curricul* OR veterinary education OR allied health personnel)**  (("medic"[All Fields] OR "medical"[All Fields] OR "medicalization"[MeSH Terms] OR "medicalization"[All Fields] OR "medicalizations"[All Fields] OR "medicalize"[All Fields] OR "medicalized"[All Fields] OR "medicalizes"[All Fields] OR "medicalizing"[All Fields] OR "medically"[All Fields] OR "medicals"[All Fields] OR "medicated"[All Fields] OR "medication s"[All Fields] OR "medics"[All Fields] OR "pharmaceutical preparations"[MeSH Terms] OR ("pharmaceutical"[All Fields] AND "preparations"[All Fields]) OR "pharmaceutical preparations"[All Fields] OR "medication"[All Fields] OR "medications"[All Fields]) AND "educat*"[All Fields]) OR (("medic"[All Fields] OR "medical"[All Fields] OR "medicalization"[MeSH Terms] OR "medicalization"[All Fields] OR "medicalizations"[All Fields] OR "medicalize"[All Fields] OR "medicalized"[All Fields] OR "medicalizes"[All Fields] OR "medicalizing"[All Fields] OR "medically"[All Fields] OR "medicals"[All Fields] OR "medicated"[All Fields] OR "medication s"[All Fields] OR "medics"[All Fields] OR "pharmaceutical preparations"[MeSH Terms] OR ("pharmaceutical"[All Fields] AND "preparations"[All Fields]) OR "pharmaceutical preparations"[All Fields] OR "medication"[All Fields] OR "medications"[All Fields]) AND "train*"[All Fields]) OR ("preceptorship"[MeSH Terms] OR "preceptorship"[All Fields] OR ("medical"[All Fields] AND "field"[All Fields] AND "training"[All Fields]) OR "medical field training"[All Fields]) OR (("medic"[All Fields] OR "medical"[All Fields] OR "medicalization"[MeSH Terms] OR "medicalization"[All Fields] OR "medicalizations"[All Fields] OR "medicalize"[All Fields] OR "medicalized"[All Fields] OR "medicalizes"[All Fields] OR "medicalizing"[All Fields] OR "medically"[All Fields] OR "medicals"[All Fields] OR "medicated"[All Fields] OR "medication s"[All Fields] OR "medics"[All Fields] OR "pharmaceutical preparations"[MeSH Terms] OR ("pharmaceutical"[All Fields] AND "preparations"[All Fields]) OR "pharmaceutical preparations"[All Fields] OR "medication"[All Fields] OR "medications"[All Fields]) AND "school*"[All Fields]) OR (("medic"[All Fields] OR "medical"[All Fields] OR "medicalization"[MeSH Terms] OR "medicalization"[All Fields] OR "medicalizations"[All Fields] OR "medicalize"[All Fields] OR "medicalized"[All Fields] OR "medicalizes"[All Fields] OR "medicalizing"[All Fields] OR "medically"[All Fields] OR "medicals"[All Fields] OR "medicated"[All Fields] OR "medication s"[All Fields] OR "medics"[All Fields] OR "pharmaceutical preparations"[MeSH Terms] OR ("pharmaceutical"[All Fields] AND "preparations"[All Fields]) OR "pharmaceutical preparations"[All Fields] OR "medication"[All Fields] OR "medications"[All Fields]) AND "intern*"[All Fields]) OR (("medic"[All Fields] OR "medical"[All Fields] OR "medicalization"[MeSH Terms] OR "medicalization"[All Fields] OR "medicalizations"[All Fields] OR "medicalize"[All Fields] OR "medicalized"[All Fields] OR "medicalizes"[All Fields] OR "medicalizing"[All Fields] OR "medically"[All Fields] OR "medicals"[All Fields] OR "medicated"[All Fields] OR "medication s"[All Fields] OR "medics"[All Fields] OR "pharmaceutical preparations"[MeSH Terms] OR ("pharmaceutical"[All Fields] AND "preparations"[All Fields]) OR "pharmaceutical preparations"[All Fields] OR "medication"[All Fields] OR "medications"[All Fields]) AND "residen*"[All Fields]) OR (("medic"[All Fields] OR "medical"[All Fields] OR "medicalization"[MeSH Terms] OR "medicalization"[All Fields] OR "medicalizations"[All Fields] OR "medicalize"[All Fields] OR "medicalized"[All Fields] OR "medicalizes"[All Fields] OR "medicalizing"[All Fields] OR "medically"[All Fields] OR "medicals"[All Fields] OR "medicated"[All Fields] OR "medication s"[All Fields] OR "medics"[All Fields] OR "pharmaceutical preparations"[MeSH Terms] OR ("pharmaceutical"[All Fields] AND "preparations"[All Fields]) OR "pharmaceutical preparations"[All Fields] OR "medication"[All Fields] OR "medications"[All Fields]) AND "student*"[All Fields]) OR (("dental health services"[MeSH Terms] OR ("dental"[All Fields] AND "health"[All Fields] AND "services"[All Fields]) OR "dental health services"[All Fields] OR "dental"[All Fields] OR "dentally"[All Fields] OR "dentals"[All Fields]) AND "student*"[All Fields]) OR (("nursing"[MeSH Terms] OR "nursing"[All Fields] OR "nursings"[All Fields] OR "nursing"[MeSH Subheading] OR "nursing s"[All Fields]) AND "student*"[All Fields]) OR (("pharmacie"[All Fields] OR "pharmacies"[MeSH Terms] OR "pharmacies"[All Fields] OR "pharmacy"[MeSH Terms] OR "pharmacy"[All Fields] OR "pharmacy s"[All Fields]) AND "student*"[All Fields]) OR (("veterinary"[MeSH Subheading] OR "veterinary"[All Fields]) AND "student*"[All Fields]) OR (("ambulatory care facilities"[MeSH Terms] OR ("ambulatory"[All Fields] AND "care"[All Fields] AND "facilities"[All Fields]) OR "ambulatory care facilities"[All Fields] OR "clinic"[All Fields] OR "clinic s"[All Fields] OR "clinical"[All Fields] OR "clinically"[All Fields] OR "clinicals"[All Fields] OR "clinics"[All Fields]) AND "education*"[All Fields]) OR (("ambulatory care facilities"[MeSH Terms] OR ("ambulatory"[All Fields] AND "care"[All Fields] AND "facilities"[All Fields]) OR "ambulatory care facilities"[All Fields] OR "clinic"[All Fields] OR "clinic s"[All Fields] OR "clinical"[All Fields] OR "clinically"[All Fields] OR "clinicals"[All Fields] OR "clinics"[All Fields]) AND "train*"[All Fields]) OR (("ambulatory care facilities"[MeSH Terms] OR ("ambulatory"[All Fields] AND "care"[All Fields] AND "facilities"[All Fields]) OR "ambulatory care facilities"[All Fields] OR "clinic"[All Fields] OR "clinic s"[All Fields] OR "clinical"[All Fields] OR "clinically"[All Fields] OR "clinicals"[All Fields] OR "clinics"[All Fields]) AND "intern*"[All Fields]) OR (("ambulatory care facilities"[MeSH Terms] OR ("ambulatory"[All Fields] AND "care"[All Fields] AND "facilities"[All Fields]) OR "ambulatory care facilities"[All Fields] OR "clinic"[All Fields] OR "clinic s"[All Fields] OR "clinical"[All Fields] OR "clinically"[All Fields] OR "clinicals"[All Fields] OR "clinics"[All Fields]) AND "residen*"[All Fields]) OR (("ambulatory care facilities"[MeSH Terms] OR ("ambulatory"[All Fields] AND "care"[All Fields] AND "facilities"[All Fields]) OR "ambulatory care facilities"[All Fields] OR "clinic"[All Fields] OR "clinic s"[All Fields] OR "clinical"[All Fields] OR "clinically"[All Fields] OR "clinicals"[All Fields] OR "clinics"[All Fields]) AND "clerk*"[All Fields]) OR (("education"[MeSH Subheading] OR "education"[All Fields] OR "teaching"[All Fields] OR "teaching"[MeSH Terms] OR "teaches"[All Fields] OR "teach"[All Fields] OR "teachings"[All Fields] OR "teaching s"[All Fields]) AND "round*"[All Fields]) OR (("dental health services"[MeSH Terms] OR ("dental"[All Fields] AND "health"[All Fields] AND "services"[All Fields]) OR "dental health services"[All Fields] OR "dental"[All Fields] OR "dentally"[All Fields] OR "dentals"[All Fields]) AND "education*"[All Fields]) OR (("pharmacie"[All Fields] OR "pharmacies"[MeSH Terms] OR "pharmacies"[All Fields] OR "pharmacy"[MeSH Terms] OR "pharmacy"[All Fields] OR "pharmacy s"[All Fields]) AND "education*"[All Fields]) OR (("pharmacie"[All Fields] OR "pharmacies"[MeSH Terms] OR "pharmacies"[All Fields] OR "pharmacy"[MeSH Terms] OR "pharmacy"[All Fields] OR "pharmacy s"[All Fields]) AND "residen*"[All Fields]) OR (("nursing"[MeSH Terms] OR "nursing"[All Fields] OR "nursings"[All Fields] OR "nursing"[MeSH Subheading] OR "nursing s"[All Fields]) AND "education*"[All Fields]) OR (("paramedic s"[All Fields] OR "paramedical"[All Fields] OR "paramedicals"[All Fields] OR "paramedics"[MeSH Terms] OR "paramedics"[All Fields] OR "paramedic"[All Fields] OR "emergency medical technicians"[MeSH Terms] OR ("emergency"[All Fields] AND "medical"[All Fields] AND "technicians"[All Fields]) OR "emergency medical technicians"[All Fields]) AND "education*"[All Fields]) OR (("paramedic s"[All Fields] OR "paramedical"[All Fields] OR "paramedicals"[All Fields] OR "paramedics"[MeSH Terms] OR "paramedics"[All Fields] OR "paramedic"[All Fields] OR "emergency medical technicians"[MeSH Terms] OR ("emergency"[All Fields] AND "medical"[All Fields] AND "technicians"[All Fields]) OR "emergency medical technicians"[All Fields]) AND "education*"[All Fields]) OR (("paramedic s"[All Fields] OR "paramedical"[All Fields] OR "paramedicals"[All Fields] OR "paramedics"[MeSH Terms] OR "paramedics"[All Fields] OR "paramedic"[All Fields] OR "emergency medical technicians"[MeSH Terms] OR ("emergency"[All Fields] AND "medical"[All Fields] AND "technicians"[All Fields]) OR "emergency medical technicians"[All Fields]) AND "education*"[All Fields]) OR (("physical therapy modalities"[MeSH Terms] OR ("physical"[All Fields] AND "therapy"[All Fields] AND "modalities"[All Fields]) OR "physical therapy modalities"[All Fields] OR "physiotherapies"[All Fields] OR "physiotherapy"[All Fields]) AND "education*"[All Fields]) OR (("physical therapists"[MeSH Terms] OR ("physical"[All Fields] AND "therapists"[All Fields]) OR "physical therapists"[All Fields] OR "physiotherapist"[All Fields] OR "physiotherapists"[All Fields] OR "physiotherapist s"[All Fields]) AND "education*"[All Fields]) OR (("emergency medical services"[MeSH Terms] OR ("emergency"[All Fields] AND "medical"[All Fields] AND "services"[All Fields]) OR "emergency medical services"[All Fields]) AND "educat*"[All Fields]) OR "curricul*"[All Fields] OR ("education, veterinary"[MeSH Terms] OR ("education"[All Fields] AND "veterinary"[All Fields]) OR "veterinary education"[All Fields] OR ("veterinary"[All Fields] AND "education"[All Fields])) OR ("allied health personnel"[MeSH Terms] OR ("allied"[All Fields] AND "health"[All Fields] AND "personnel"[All Fields]) OR "allied health personnel"[All Fields])  **Translations**  **medical:** "medic"[All Fields] OR "medical"[All Fields] OR "medicalization"[MeSH Terms] OR "medicalization"[All Fields] OR "medicalizations"[All Fields] OR "medicalize"[All Fields] OR "medicalized"[All Fields] OR "medicalizes"[All Fields] OR "medicalizing"[All Fields] OR "medically"[All Fields] OR "medicals"[All Fields] OR "medicated"[All Fields] OR "medication's"[All Fields] OR "medics"[All Fields] OR "pharmaceutical preparations"[MeSH Terms] OR ("pharmaceutical"[All Fields] AND "preparations"[All Fields]) OR "pharmaceutical preparations"[All Fields] OR "medication"[All Fields] OR "medications"[All Fields]  **medical:** "medic"[All Fields] OR "medical"[All Fields] OR "medicalization"[MeSH Terms] OR "medicalization"[All Fields] OR "medicalizations"[All Fields] OR "medicalize"[All Fields] OR "medicalized"[All Fields] OR "medicalizes"[All Fields] OR "medicalizing"[All Fields] OR "medically"[All Fields] OR "medicals"[All Fields] OR "medicated"[All Fields] OR "medication's"[All Fields] OR "medics"[All Fields] OR "pharmaceutical preparations"[MeSH Terms] OR ("pharmaceutical"[All Fields] AND "preparations"[All Fields]) OR "pharmaceutical preparations"[All Fields] OR "medication"[All Fields] OR "medications"[All Fields]  **medical field training:** "preceptorship"[MeSH Terms] OR "preceptorship"[All Fields] OR ("medical"[All Fields] AND "field"[All Fields] AND "training"[All Fields]) OR "medical field training"[All Fields]  **medical:** "medic"[All Fields] OR "medical"[All Fields] OR "medicalization"[MeSH Terms] OR "medicalization"[All Fields] OR "medicalizations"[All Fields] OR "medicalize"[All Fields] OR "medicalized"[All Fields] OR "medicalizes"[All Fields] OR "medicalizing"[All Fields] OR "medically"[All Fields] OR "medicals"[All Fields] OR "medicated"[All Fields] OR "medication's"[All Fields] OR "medics"[All Fields] OR "pharmaceutical preparations"[MeSH Terms] OR ("pharmaceutical"[All Fields] AND "preparations"[All Fields]) OR "pharmaceutical preparations"[All Fields] OR "medication"[All Fields] OR "medications"[All Fields]  **medical:** "medic"[All Fields] OR "medical"[All Fields] OR "medicalization"[MeSH Terms] OR "medicalization"[All Fields] OR "medicalizations"[All Fields] OR "medicalize"[All Fields] OR "medicalized"[All Fields] OR "medicalizes"[All Fields] OR "medicalizing"[All Fields] OR "medically"[All Fields] OR "medicals"[All Fields] OR "medicated"[All Fields] OR "medication's"[All Fields] OR "medics"[All Fields] OR "pharmaceutical preparations"[MeSH Terms] OR ("pharmaceutical"[All Fields] AND "preparations"[All Fields]) OR "pharmaceutical preparations"[All Fields] OR "medication"[All Fields] OR "medications"[All Fields]  **medical:** "medic"[All Fields] OR "medical"[All Fields] OR "medicalization"[MeSH Terms] OR "medicalization"[All Fields] OR "medicalizations"[All Fields] OR "medicalize"[All Fields] OR "medicalized"[All Fields] OR "medicalizes"[All Fields] OR "medicalizing"[All Fields] OR "medically"[All Fields] OR "medicals"[All Fields] OR "medicated"[All Fields] OR "medication's"[All Fields] OR "medics"[All Fields] OR "pharmaceutical preparations"[MeSH Terms] OR ("pharmaceutical"[All Fields] AND "preparations"[All Fields]) OR "pharmaceutical preparations"[All Fields] OR "medication"[All Fields] OR "medications"[All Fields]  **medical:** "medic"[All Fields] OR "medical"[All Fields] OR "medicalization"[MeSH Terms] OR "medicalization"[All Fields] OR "medicalizations"[All Fields] OR "medicalize"[All Fields] OR "medicalized"[All Fields] OR "medicalizes"[All Fields] OR "medicalizing"[All Fields] OR "medically"[All Fields] OR "medicals"[All Fields] OR "medicated"[All Fields] OR "medication's"[All Fields] OR "medics"[All Fields] OR "pharmaceutical preparations"[MeSH Terms] OR ("pharmaceutical"[All Fields] AND "preparations"[All Fields]) OR "pharmaceutical preparations"[All Fields] OR "medication"[All Fields] OR "medications"[All Fields]  **dental:** "dental health services"[MeSH Terms] OR ("dental"[All Fields] AND "health"[All Fields] AND "services"[All Fields]) OR "dental health services"[All Fields] OR "dental"[All Fields] OR "dentally"[All Fields] OR "dentals"[All Fields]  **nursing:** "nursing"[MeSH Terms] OR "nursing"[All Fields] OR "nursings"[All Fields] OR "nursing"[Subheading] OR "nursing's"[All Fields]  **pharmacy:** "pharmacie"[All Fields] OR "pharmacies"[MeSH Terms] OR "pharmacies"[All Fields] OR "pharmacy"[MeSH Terms] OR "pharmacy"[All Fields] OR "pharmacy's"[All Fields]  **veterinary:** "veterinary"[Subheading] OR "veterinary"[All Fields]  **clinical:** "ambulatory care facilities"[MeSH Terms] OR ("ambulatory"[All Fields] AND "care"[All Fields] AND "facilities"[All Fields]) OR "ambulatory care facilities"[All Fields] OR "clinic"[All Fields] OR "clinic's"[All Fields] OR "clinical"[All Fields] OR "clinically"[All Fields] OR "clinicals"[All Fields] OR "clinics"[All Fields]  **clinical:** "ambulatory care facilities"[MeSH Terms] OR ("ambulatory"[All Fields] AND "care"[All Fields] AND "facilities"[All Fields]) OR "ambulatory care facilities"[All Fields] OR "clinic"[All Fields] OR "clinic's"[All Fields] OR "clinical"[All Fields] OR "clinically"[All Fields] OR "clinicals"[All Fields] OR "clinics"[All Fields]  **clinical:** "ambulatory care facilities"[MeSH Terms] OR ("ambulatory"[All Fields] AND "care"[All Fields] AND "facilities"[All Fields]) OR "ambulatory care facilities"[All Fields] OR "clinic"[All Fields] OR "clinic's"[All Fields] OR "clinical"[All Fields] OR "clinically"[All Fields] OR "clinicals"[All Fields] OR "clinics"[All Fields]  **clinical:** "ambulatory care facilities"[MeSH Terms] OR ("ambulatory"[All Fields] AND "care"[All Fields] AND "facilities"[All Fields]) OR "ambulatory care facilities"[All Fields] OR "clinic"[All Fields] OR "clinic's"[All Fields] OR "clinical"[All Fields] OR "clinically"[All Fields] OR "clinicals"[All Fields] OR "clinics"[All Fields]  **clinical:** "ambulatory care facilities"[MeSH Terms] OR ("ambulatory"[All Fields] AND "care"[All Fields] AND "facilities"[All Fields]) OR "ambulatory care facilities"[All Fields] OR "clinic"[All Fields] OR "clinic's"[All Fields] OR "clinical"[All Fields] OR "clinically"[All Fields] OR "clinicals"[All Fields] OR "clinics"[All Fields]  **teaching:** "education"[Subheading] OR "education"[All Fields] OR "teaching"[All Fields] OR "teaching"[MeSH Terms] OR "teaches"[All Fields] OR "teach"[All Fields] OR "teachings"[All Fields] OR "teaching's"[All Fields]  **dental:** "dental health services"[MeSH Terms] OR ("dental"[All Fields] AND "health"[All Fields] AND "services"[All Fields]) OR "dental health services"[All Fields] OR "dental"[All Fields] OR "dentally"[All Fields] OR "dentals"[All Fields]  **pharmacy:** "pharmacie"[All Fields] OR "pharmacies"[MeSH Terms] OR "pharmacies"[All Fields] OR "pharmacy"[MeSH Terms] OR "pharmacy"[All Fields] OR "pharmacy's"[All Fields]  **pharmacy:** "pharmacie"[All Fields] OR "pharmacies"[MeSH Terms] OR "pharmacies"[All Fields] OR "pharmacy"[MeSH Terms] OR "pharmacy"[All Fields] OR "pharmacy's"[All Fields]  **nursing:** "nursing"[MeSH Terms] OR "nursing"[All Fields] OR "nursings"[All Fields] OR "nursing"[Subheading] OR "nursing's"[All Fields]  **paramedics:** "paramedic's"[All Fields] OR "paramedical"[All Fields] OR "paramedicals"[All Fields] OR "paramedics"[MeSH Terms] OR "paramedics"[All Fields] OR "paramedic"[All Fields] OR "emergency medical technicians"[MeSH Terms] OR ("emergency"[All Fields] AND "medical"[All Fields] AND "technicians"[All Fields]) OR "emergency medical technicians"[All Fields]  **paramedic:** "paramedic's"[All Fields] OR "paramedical"[All Fields] OR "paramedicals"[All Fields] OR "paramedics"[MeSH Terms] OR "paramedics"[All Fields] OR "paramedic"[All Fields] OR "emergency medical technicians"[MeSH Terms] OR ("emergency"[All Fields] AND "medical"[All Fields] AND "technicians"[All Fields]) OR "emergency medical technicians"[All Fields]  **paramedical:** "paramedic's"[All Fields] OR "paramedical"[All Fields] OR "paramedicals"[All Fields] OR "paramedics"[MeSH Terms] OR "paramedics"[All Fields] OR "paramedic"[All Fields] OR "emergency medical technicians"[MeSH Terms] OR ("emergency"[All Fields] AND "medical"[All Fields] AND "technicians"[All Fields]) OR "emergency medical technicians"[All Fields]  **physiotherapy:** "physical therapy modalities"[MeSH Terms] OR ("physical"[All Fields] AND "therapy"[All Fields] AND "modalities"[All Fields]) OR "physical therapy modalities"[All Fields] OR "physiotherapies"[All Fields] OR "physiotherapy"[All Fields]  **physiotherapist:** "physical therapists"[MeSH Terms] OR ("physical"[All Fields] AND "therapists"[All Fields]) OR "physical therapists"[All Fields] OR "physiotherapist"[All Fields] OR "physiotherapists"[All Fields] OR "physiotherapist's"[All Fields]  **emergency medical services:** "emergency medical services"[MeSH Terms] OR ("emergency"[All Fields] AND "medical"[All Fields] AND "services"[All Fields]) OR "emergency medical services"[All Fields]  **veterinary education:** "education, veterinary"[MeSH Terms] OR ("education"[All Fields] AND "veterinary"[All Fields]) OR "veterinary education"[All Fields] OR ("veterinary"[All Fields] AND "education"[All Fields])  **allied health personnel:** "allied health personnel"[MeSH Terms] OR ("allied"[All Fields] AND "health"[All Fields] AND "personnel"[All Fields]) OR "allied health personnel"[All Fields] |  |  |

Table S2A: Summary of the extracted information

| Question | Data Extracted |
| --- | --- |
| RQ1 | General information: author, year of publication, country/ site |
| RQ1.1 | General information: publication source and channel (QA5) |
| RQ2 | Geographic distribution and prevalence of articles |
| RQ3 | Content of the game: AMR, AMS, ID, CM |
| RQ4 | Context of the study: human, animal, environmental or “One Health” |
| RQ5 | Intervention: game format used (board game, card game, online etc.) |
| RQ6 | Intervention: game elements used (points, scoring, roleplay etc.) |
| RQ7 | Benefits and limitations of the intervention |
| RQ8 | Aim of the game: knowledge, attitudes, behaviour change, entertainment etc |
| RQ9 | Key message |

Table S2B: Quality assessment criteria

| # | Question | Possible answers |
| --- | --- | --- |
| QA1 | Does the paper present a detailed description of the game elements employed? | Yes (+1)  No (+0)  Partially (+0.5) |
| QA2 | Does the study present empirical results? | Yes (+1)  No ‘(+0) |
| QA3 | Are the benefits of gamification for antimicrobial stewardship addressed explicitly? | Yes (+1)  No ‘(+0) |
| QA4 | Are the limitations of the game addressed? | Yes (+1)  No ‘(+0) |
| QA5 | Has the study been published in a relevant journal or conference proceedings?   - For conferences, workshops, and symposia:      - For Journals | (+1.5) if it is ranked CORE A  (+1) if it is ranked CORE B  (+0.5) if it is ranked CORE C  (+0) if it is not in a CORE ranking.  (+2) if it is ranked Q1  (+1.5) if it is ranked Q2  (+1) if it is ranked Q3  Q4 (+0) if it has no JCR ranking.   For others: (+0) |

Table S3. All articles breakdown

|  | Author | Year Published | Location |  | Journal | Setting | Game | Sample Size |
| --- | --- | --- | --- | --- | --- | --- | --- | --- |
| 3 | Ghelfenstein-Ferreira et al. | 2021 | Paris, France | Original paper | Journal of Microbiology and Biology Education (JMBE) | Education evening (game night) | Bacteria Game KROBS Dawaa | 15 |
| 56 | Castro-Sánchez et al. | 2019 | London, United Kingdom | Original paper | Journal of Medical Internet research (JMIR) | Workshop at a conference (International Summit on Serious Health Games) | On call: Antibiotics. | 29 |
| 63 | Angharad DP | 2020 | United Kingdom | Short communication | Medical Science Educator | Once-off game session with additional (optional) tutorial for first-year medical students | Antibiotic Top Trumps | 36 |
| 88 | Valente et al. | 2009 | Porto Alegre, Brazil | Original paper | Medical Teacher | Once-off game session | no name | 78 |
| 93 | Tsopra et al. | 2020 | France | Original paper | International Journal of Medical Informatics (IJMI) |  | AntibioGame® | 57 |
| 98 | Ashiru-Oredope et al. | 2022 | Global | Original paper | MDPI Antibiotics |  |  | 74 |
|  |  |  | United Kingdom |  |  |  |  | 38 |
|  |  |  | Hungary |  |  |  |  | 1 |
|  |  |  | India |  |  |  |  | 1 |
|  |  |  | Sri Lanka |  |  |  |  | 1 |
|  |  |  | Uganda |  |  |  |  | 15 |
|  |  |  | Kenya |  |  |  |  | 7 |
|  |  |  | Ghana |  |  |  |  | 2 |
|  |  |  | Nigeria |  |  |  |  | 3 |
|  |  |  | Sierra Leone |  |  |  |  | 3 |
|  |  |  | Eswatini |  |  |  |  | 1 |
|  |  |  | Malawi |  |  |  |  | 1 |
|  |  |  | Fiji |  |  |  |  | 1 |

Part 2:

| Author | Sector of Study | Game Content | Student type | Benefits | Limitations |
| --- | --- | --- | --- | --- | --- |
| Ghelfenstein-Ferreira et al. | Human Health | CM, ABX (20 bacteria's traits, 20 microorganisms' traits and means of transmission, and the usage of antibiotics.) | Medical residents specializing in infectious disease or clinical microbiology | Comment: significant educational contribution, favorable and meaningful positive interactions between participants.  Education: increase in scores during the evening regarding questions addressed by the games. | Bacteria game -- independent gameplay or under supervision of CM; Krobs -- independent gameplay or supervision of CM; Dawaa -- needs supervision of ID specialist. Low number of participants (15) does not conclude that the use of games does not have an impact on learning in general. One social event does not mean that the increase in scores is attributable to the game. Study pool are students who already have knowledge in ID and CM. |
| Castro-Sánchez et al. | Human Health | AMS, BEHAVIOUR (Promoting superior antimicrobial practices while acknowledging that these practices' ideal attributes may depend on a range of interrelated professional, clinical, and organizational circumstances as well as patient expectations) | Consultant physicians from different clinical specialties; doctoral and postdoctoral researchers with projects focused on simulation, games, or virtual environments; AMR researchers and clinicians; experts in digital intervention implementation; games developers; behavioural researchers with interests in game-based interventions | Comment: fosters excellent antimicrobial behaviours -- multidisciplinary approach and includes management of patient expectations | Does not aim to teach about specific antibiotics, appropriateness, or effectiveness in the treatment of an infection |
| Angharad DP | Human Health | CM, ABX (Learning about various gram-stained microorganisms and what medications can be used to combat them) | First year medical students | Education: little difference before and after playing the game. Themes were fun, content of the cards were helpful, and pictures were nice, a fun way to apply knowledge, a good revision aid. | Education: fun but not useful to learn, too fast paced, wasn't paying attention to card details rather just the stats of the card. Suggestion that playing with cards at intervals over weeks could result better retention of information. |
| Valente et al. | Human Health | CM, ABX (basic microbiology and antibiotics mode of action) | Medical and pharmacy students | Education: significant increase in the number of right answers, decrease in number of unknown answers. Comment: interesting, with clear design and improved knowledge about the subject, important way of enhancing learning, literature appropriate. Valuable for improvement of intellectual skills. | Education: does not provide acquisition of practical/ manual skills. Independent game, no need for supervision. |
| Tsopra et al. | Human Health | AMS, PRESCRIBING (Public health interventions - patient interventions, antibiotic prescription, and naming) | Medical students having completed 2 years of medical school | Education: attractive, fun, and appropriate for learning about antibiotics. A good revision aid. | Education: there is a limited bank of questions (10 cases only), the topic did not extend to other specialities, topics related to AMR such as mechanisms of bacteria resistance were excluded, medical context was only for GP office and excluded the management opportunities for things like ICU |
| Ashiru-Oredope et al. | Human Health | AMR, AMS, IPC, ABX (1) introduction to AMR and AMS, (2) appropriate use of antimicrobial agents, (3) infection prevention and control and (4) stewardship and surveillance. | Out of 74 respondents, only 7 were students | Comment: the game was entertaining and enriching, interesting and straight forward. Incorporates the One Health approach partially, and both high-income countries and low-middle income countries. | Comment: the demonstration was rushed therefore unable to get a real feel of the game, it was played for a very short time. Cross talking among participants make it difficult for others to respond and the facilitators talked too much. |

Part 3:

| Author | Type of Game | Game Elements | Aim of the Game | Description of the Intervention | Gameplay evaluation |
| --- | --- | --- | --- | --- | --- |
| Ghelfenstein-Ferreira et al. | Board Game 2 Card Games | Points  Scoring | Entertainment Improve knowledge Address prescribing practices | The board game and 2 card games were played at a game night for residents. | Played for 30 mins |
| Castro-Sánchez et al. | Mobile case-based game | Story  Roleplay | Entertainment Address prescribing practices Change behavior | The game resembles clinical practice: there are virtual patients that present with a condition, and the student must use and employ diagnostic skills and optimal behaviors that one should be familiar with based on the established antibiotic guidelines in the country (United Kingdom). | 2-hour workshop. On call: Antibiotics developed in 2015, with 4000 downloads. Not an evaluation of the game per says; but rather a workshop setting to debate the limitations and gaps. |
| Angharad DP | Card Game | Scoring | Improve knowledge Change behavior | The game resembles an existing game called Top trumps; it has been tailored to information related to antibiotics. The cards had details about commonly used antibiotics with scores against the microorganisms, toxicity, and administration routes. It also had an additional fun/ useful fact. | Played for 10 mins; pre-test and post-test with additional optional lecture |
| Valente et al. | Board Game | Leaderboard | Improve knowledge | The game is an illustrated board game. Layers must move their token across the board game, they pass over squares in which there are antibiotic name, and "pick up a card" squares. The cards detail a bacterium and entails information on whether it is susceptible/ resistant or intermediate resistance to the antibiotic card. In addition, there are also cards where there are textbook questions. The player who picks up the card must answer the questions. | Between 40 mins and 90 mins; pre-test and post-test with additional lecture given a week prior to the intervention |
| Tsopra et al. | Online Game (Case-based game) -- Serious game -- Interactive | Challenge Mascot (Antibioman) Avatar with badges Hints Feedback Rewards Points Level and progress bars Leaderboard Immersion in the real world | Address prescribing practices Change behavior | This game is a case-based game for teaching students about antibiotics in primary care. The player plays the role of doctor and solves the case using clinical reasoning. | Each session began with a presentation of the game. Three clinical cases were played by the students before an evaluation and comparison of the scores took place. |
| Ashiru-Oredope et al. | Online Board Game | Points | Improve knowledge Change behavior | The game is an online board game similar to the "snakes and ladders" concept. | The online game was played by over 100 students in 23 different countries on two occasions (August 2021 and November 2021) and was played using Zoom. The game lasted 45 minutes. Only 74 participants completed the feedback form upon completion of the intervention. Of which, only 7 participants who responded were students. |
